# The Impact of Trimethylamine N-Oxide and Coronary Microcirculatory Dysfunction on Outcomes following ST-Elevation Myocardial Infarction

**DOI:** 10.3390/jcdd10050197

**Published:** 2023-04-29

**Authors:** Ali Aldujeli, Riddhi Patel, Ingrida Grabauskyte, Anas Hamadeh, Austeja Lieponyte, Vacis Tatarunas, Hussein Khalifeh, Kasparas Briedis, Vilius Skipskis, Montazar Aldujeili, Dalia Jarasuniene, Sumit Rana, Ramunas Unikas, Ayman Haq

**Affiliations:** 1 Faculty of Medicine, Lithuanian University of Health Sciences, 44307 Kaunas, Lithuania; 2 HCA Medical City Healthcare UNT-TCU Graduate Medical Education Program, Arlington, TX 76015, USA; 3 Heart & Vascular Specialists of North Texas, Arlington, TX 76014, USA; 4 Kreiskrankenhaus Rotenburg an der Fulda, 36199 Rotenburg an der Fulda, Germany; 5 Faculty of Medicine, University of Brescia, 25121 Brescia, Italy; 6 Seamen’s Branch, Department of Cardiology, Klaipeda University Hospital, 92288 Klaipeda, Lithuania; 7 Thorndale Medical Clinic, D05 DX09 Dublin, Ireland; 8 Abbott Northwestern Hospital, Minneapolis, MN 55407, USA; 9 Minneapolis Heart Institute Foundation, Minneapolis, MN 55407, USA

**Keywords:** coronary microcirculatory dysfunction, CMD, atrial fibrillation, coronary flow reserve, CFR, index of microvascular resistance, IMR, TMAO

## Abstract

Introduction: Persistent coronary microcirculatory dysfunction (CMD) and elevated trimethylamine N-oxide (TMAO) levels after ST-elevation myocardial infarction (STEMI) may drive negative structural and electrical cardiac remodeling, resulting in new-onset atrial fibrillation (AF) and a decrease in left ventricular ejection fraction (LVEF). Aims: TMAO and CMD are investigated as potential predictors of new-onset AF and left ventricular remodeling following STEMI. Methods: This prospective study included STEMI patients who had primary percutaneous coronary intervention (PCI) followed by staged PCI three months later. Cardiac ultrasound images were obtained at baseline and after 12 months to assess LVEF. Coronary flow reserve (CFR), and index of microvascular resistance (IMR) were assessed using the coronary pressure wire during the staged PCI. Microcirculatory dysfunction was defined as having an IMR value ≥25 U and CFR value <2.5 U. Results: A total of 200 patients were included in the study. Patients were categorized according to whether or not they had CMD. Neither group differed from the other with regards to known risk factors. Despite making up only 40.5% of the study population, females represented 67.4% of the CMD group *p* < 0.001. Similarly, CMD patients had a much higher prevalence of diabetes than those without CMD (45.7% vs. 18.2%; *p* < 0.001). At the one-year follow-up, the LVEF in the CMD group had decreased to significantly lower levels than those in the non-CMD group (40% vs. 50%; *p* < 0.001), whereas it had been higher in the CMD group at baseline (45% vs. 40%; *p* = 0.019). Similarly, during the follow-up, the CMD group had a greater incidence of AF (32.6% vs. 4.5%; *p* < 0.001). In the adjusted multivariable analysis, the IMR and TMAO were associated with increased odds of AF development (OR: 1.066, 95% CI: 1.018–1.117, *p* = 0.007), and (OR: 1.290, 95% CI: 1.002–1.660, *p* = 0.048), respectively. Similarly, elevated levels of IMR and TMAO were linked with decreased odds of LVEF improvement, while higher CFR values are related to a greater likelihood of LVEF improvement. Conclusions: CMD and elevated TMAO levels were highly prevalent three months after STEMI. Patients with CMD had an increased incidence of AF and a lower LVEF 12 months after STEMI.

## 1. Introduction

New-onset atrial fibrillation (AF) and left ventricular systolic function deterioration remains a prevalent consequence of ST-elevation myocardial infarction (STEMI) [1]. Despite the well-established prognostic implications new-onset AF and adverse cardiac remodeling after acute coronary syndrome, there is a lack of a reliable predictive tool for risk classification [2].

Structural and electrical cardiac remodeling plays a crucial role in the development of AF in patients who have had an acute myocardial infarction (AMI) [3]. Left atrial volume index (LAVI) and left ventricular ejection fraction (LVEF) have been found to be useful in predicting new-onset AF [4]. Coronary microcirculatory dysfunction (CMD) appears to play a key role adverse cardiac structural remodeling and may also play a role in adverse cardiac electrical remodeling [5].

Trimethylamine N-oxide (TMAO), a novel biomarker, may also play a role in cardiac electric remodeling. Trimethylamine is produced from the breakdown of dietary L-carnitine, choline, and lecithin in the human gut and is then oxidized in the liver to form TMAO [6]. Atherosclerosis, congestive heart failure, acute myocardial infarction, and cardiovascular mortality are all linked to elevated TMAO levels [6]. A 2018 animal study found that injecting TMAO into four major atrial ganglionated plexuses of healthy dogs led to increased neural activity, electrical remodeling, and activation of the proinflammatory NF-κB pathway, all of which contributed to worsening autonomic remodeling and the development of AF [7]. Three years later, a study on rat models revealed that elevated circulating TMAO levels are associated with the worsening of cardiac systolic function [8].

The current study aims to investigate the role of coronary microcirculatory dysfunction and TMAO in predicting new-onset AF and left ventricular systolic function in patients who have had a STEMI.

## 2. Methods

### 2.1. Study Design

This was a prospective single-center cohort study conducted in the Hospital of the Lithuanian University of Health Sciences Kaunas Clinics which covers four out of ten administrative regions in the Republic of Lithuania. The study was carried out in agreement with the declaration of Helsinki and approved by the Kaunas Regional Biomedical Research Ethics Committee (Kaunas, Republic of Lithuania, nr: BE-2–5); informed consent was obtained from all study participants.

### 2.2. Study Inclusion and Exclusion Criteria

The study included patients aged 40 years or older with STEMI who received dual antiplatelet therapy (acetylsalicylic acid 300 mg and ticagrelor 180 mg or clopidogrel 600 mg) and successfully underwent primary percutaneous coronary intervention (PCI) of the culprit vessel, followed by a staged PCI for non-culprit disease three months later.

Patients were excluded from the study if they had a history of atrial fibrillation/flutter, a concomitant illness affecting blood counts or inflammatory biomarkers, or significant comorbid disease (liver disease, end-stage renal failure, solid organ malignancy). Patients with severe valvular heart disease were excluded due to the risk of ventricular fibrillation with hyperemia and those with coronary artery bypass grafts were excluded due to altered coronary circulation [9]. Patients who underwent primary fibrinolysis, had a contrast media allergy or were unable to tolerate adenosine triphosphate were also excluded.

### 2.3. Blood Sampling and Laboratory Testing

Venous blood was collected from all study participants at admission, and at a three-month follow-up. Complete blood counts, serial troponin I levels, Erythrocyte sedimentation rate (ESR), B-type natriuretic peptide (BNP), high sensitivity reactive protein, and lipid levels were obtained as part of routine clinical care. The blood samples were centrifuged appropriately to obtain plasma samples, which were stored at −80 °C. TMAO ELISA (enzyme-linked immunosorbent assay) kit (Bioassay Technology Laboratory, Shanghai, China) was utilized to measure plasma TMAO levels at 3-month follow-up.

### 2.4. Coronary Physiology Assessment

Coronary flow reserve (CFR), fractional flow reserve (FFR) and index of microvascular resistance (IMR) were assessed using the CoroFlow system (Coroventis Research AB, Uppsala, Sweden). After undergoing staged PCI, nitroglycerin was administered through the intracoronary catheter and a calibrated coronary physiology wire (Pressure Wire X; Abbott Vascular, Santa Clara, CA, USA) was equalized to the guide catheter pressure at the sinus of the aorta. The coronary physiology wire was then advanced to the distal two-thirds of the left anterior descending artery. Three milliliters of normal saline was administered through the guiding catheter in triplicates during rest and during maximal hyperemia, which was induced by injecting intracoronary adenosine.

### 2.5. Data Collection

Data collected included patient demographics, medical history, clinical course, laboratory values, angiographic characteristics and follow-up data were recorded. Linear measurements of the left atrial chamber, left atrial volume (LAV), LAVI, and LVEF were obtained according to the recommendations of the European Association of Cardiovascular Imaging (EACVI), which was evaluated through obtaining two-dimensional and three-dimensional images using an ultrasound device (EPIQ 7, Phillips Ultrasound, Inc. Washington, DC, USA) over a 24-h period, and with a one-year follow-up [10].

### 2.6. Study Endpoints

The primary endpoint was the existence of atrial fibrillation twelve months following an ST-elevation myocardial infarction. The secondary endpoint was the recognition of improved left ventricular systolic function following a year of echocardiographic monitoring. Any interactions between CMD, new-onset AF and change in LVEF were also examined.

### 2.7. Definition

STEMI was defined according to the fourth universal definition of myocardial infarction [11]. Door-to-wire time was defined as the time (in minutes) from the first medical contact at the facility to the time of advancement of the PCI wire. Dyslipidemia was defined as a fasting total cholesterol level of more than 70 mg/dl (1.8 mmol/L) or the use of lipid-lowering medications [12]. Hypertension was defined as blood pressure greater than or equal to 140/90 mmHg or the use of blood pressure-lowering medication [13]. Diabetes mellitus was defined as a fasting plasma glucose level ≥ 7.0 mmol/L, or the use of blood glucose-lowering medication [14]. Successful PCI was defined as the implantation of a second-generation drug-eluting stent resulting in the reduction of a coronary artery lesion to less than 20% with the restoration of coronary blood flow. The normal values for FFR, CFR and IMR were defined as > 0.80, ≥ 2.5 and < 25 U respectively [15]. Microcirculatory dysfunction was defined as having an IMR value ≥ 25 U and a CFR value < 2.5 U [16]. Renal function was assessed by calculating the glomerular filtration rate using the Cockcroft–Gault Equation.

### 2.8. Statistical Analysis

Continuous variables were skewed and are presented as median [quartile 1, quartile 3]. Categorical variables are presented as frequency and percentage. Differences in patient and clinical characteristics between those with confirmed CMD versus the non-CMD were assessed via the Wilcoxon rank sum test and chi-square or Fisher’s exact test, as appropriate. Differences in the outcomes between both study groups were assessed via chi-square or Fisher’s exact test, as appropriate. We used stepwise selection (backward and forward selection) to create multivariable logistic regression models to investigate factors identified as having a significant association with primary and secondary outcomes. We adjusted for potential confounders (i.e., age, gender, and BMI) in the multivariable regression model designed to investigate independent predictors of AF. A probability level of *p* < α (α—significance level; α = 0.05) was taken as statistically significant. Statistical analysis was performed using IBM SPSS Statistics for Windows, Version 29.0 (IBM Corp., Armonk, NY, USA, 2022)

## 3. Results

Two hundred patients were included, of which 46 patients (23%) had CMD, while 154 patients (77%) did not. There were no differences in age, BMI, STEMI location, or risk factors between the two groups (Table 1). Despite accounting for a minority (40.5%) of the study population, females were over-represented in the CMD group (67.4%, *p* < 0.001). Similarly, diabetic patients were more prevalent in the CMD group than in the non-CMD group (45.7% vs. 18.2%; *p* < 0.001) (Table 1).

Furthermore, there were no differences in on-admission blood laboratory tests (complete blood count with differential, lipid panel, and troponin levels) between the two groups, except for creatinine clearance which was lower in the CMD group (36.6 mL/min vs. 40.9 mL/min; *p* = 0.001) (Table 2).

Total cholesterol, low-density lipoprotein cholesterol, high-density lipoprotein cholesterol and triglyceride levels all improved at three months compared to admission values (Table 2). High sensitivity CRP (4.67 mg/L vs. 4.02 mg/L; *p* = 0.021) and ESR (15.5 mm/h vs. 12.5 mm/h; *p* = 0.04) were higher in the CMD group after three months. Similarly, the CMD group had higher levels of BNP and TMAO at three months than the non-CMD group. (73.0 ng/L vs. 37.0 ng/L; *p* < 0.001) and (5.28 µM vs. 2.41 µM; *p* < 0.001), respectively (Table 2).

There was also no statistically significant difference between the two groups in terms of angiographic (pre/post PCI TIMI flow, culprit vessel, and number of diseased vessels) or echocardiographic (left atrial diameter, volume, volume index, etc.) parameters (Table 3). In contrast, post-PCI LVEF was higher in the CMD group when compared to the non-CMD group (45% vs. 40%; *p* = 0.019), nonetheless, this was short-lived, as the LVEF in the CMD group deteriorated at one year compared to the non-CMD group (40% vs. 50%; *p* < 0.001) (Table 3).

At one year, the incidence of new-onset AF was higher in the CMD group (32.6% vs. 4.5%; *p* < 0.001). However, those in the CMD group had lower rates of LVEF improvement (29.5% vs. 92.8%; *p* < 0.001) (Table 4).

**Figure 1:**
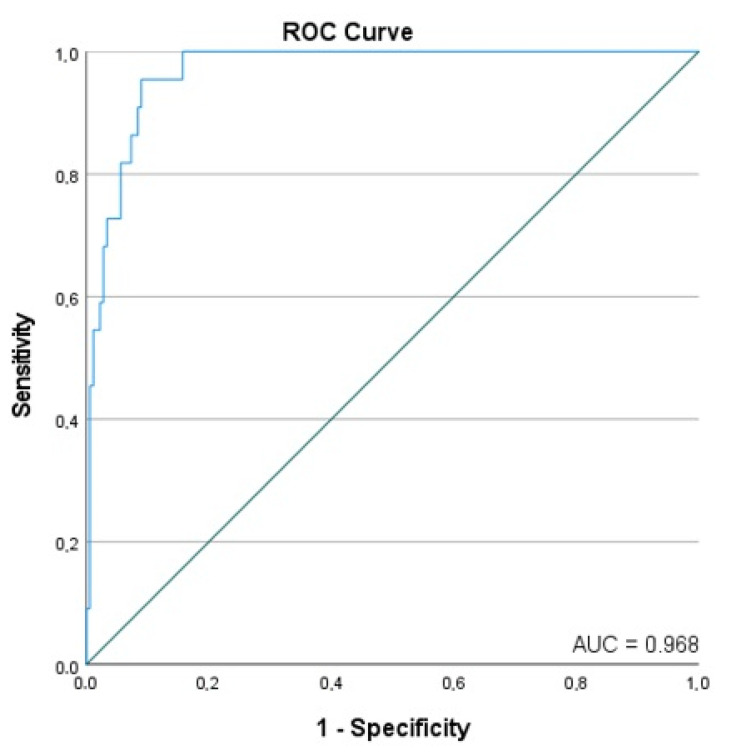
Receiver operating characteristic with the associated area under the curve (AUC) for the model of new-onset of atrial fibrillation within 12 months after STEMI.

**Figure 2:**
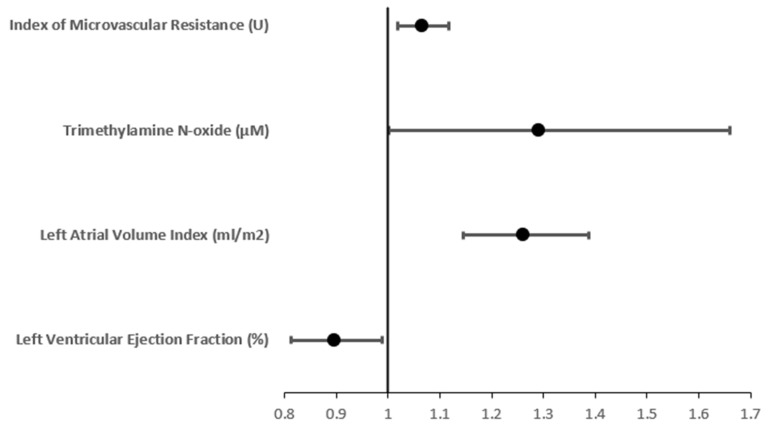
Forest plot detailing the odds ratio and 95% confidence interval between the post PCI left ventricular ejection fraction, post PCI left atrial volume index, three-month trimethylamine N-oxide level and three-month index of microvascular resistance.

On their own, IMR and TMAO yielded a receiver operating characteristic (ROC) area under the curve (AUC) of 0.763 and 0.669 respectively, indicating good predictive ability for new-onset AF (Table 5). In the adjusted binary logistic multivariable analysis, TMAO, IMR, LAVI, and LVEF had an ROC AUC of 0.968, indicating excellent predictive ability (Figure 1). In this model, after adjusting for age, gender and BMI, we have found that the IMR, LAVI, and TMAO were associated with increased odds of new-onset AF (OR: 1.066, 95% CI: 1.018–1.117, *p* = 0.007), (OR: 1.261, 95% CI: 1.146–1.387, *p* < 0.001), and (OR: 1.290, 95% CI: 1.002–1.660, *p* = 0.048), respectively (Table 6, Figure 2). On the contrary, each percentage point increase in LVEF was associated with a decreased odds of new-onset AF (OR: 0.897, 95% CI: 0.813–0.989, *p* = 0.029) (Table 6, Figure 2).

**Figure 3:**
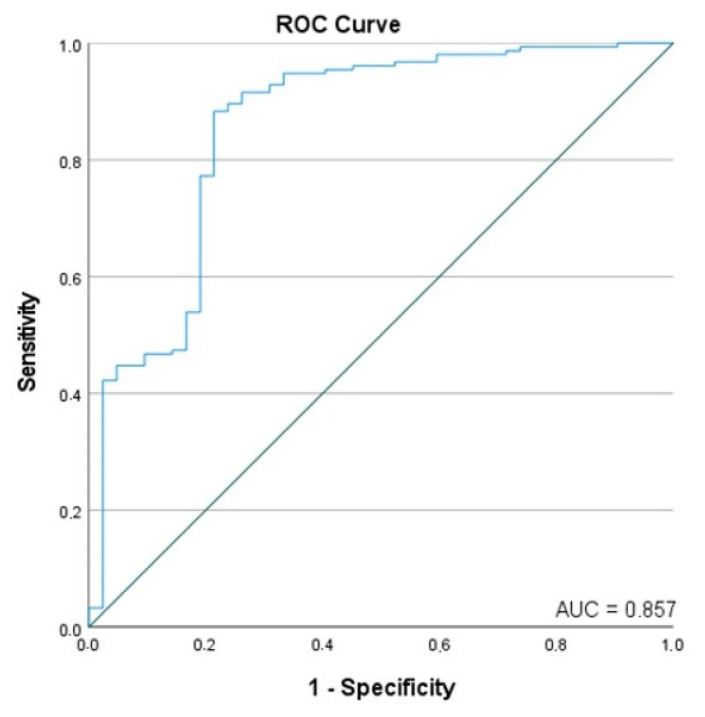
Receiver operating characteristic with the associated area under the curve (AUC) for the improvement in left ventricular ejection fraction within 12 months after STEMI.

Finally, we investigated the independent predictors of LVEF improvement at 12 months. A multivariate model incorporating TMAO, IMR, and CFR showed an ROC AUC of 0.857, indicating a high degree of predictive ability (Figure 3). In this model, we found lower odds of LVEF improvement with an elevated IMR (OR: 0.941, 95% CI: 0.910–0.973, *p* < 0.001) or an elevated TMAO (OR: 0.777, 95% CI: 0.665–0.909, *p* = 0.002) (Table 7). Conversely, higher CFR values are associated with higher odds of LVEF improvement (OR: 2.438, 95% CI: 1.064–5.585, *p* = 0.035) (Table 7).

## 4. Discussion

Two hundred patients presenting with STEMI who underwent primary PCI were enrolled in this study. At three months, the presence of CMD was assessed and at 12 months the incidence of new-onset AF and change in LVEF were assessed. Patients with CMD were more likely to have worse renal function on admission, higher BNP and higher TMAO at three months, and lower LVEF at 12 months. In addition to well-established factors such as LVEF and LAVI, this study found that the presence of CMD and a higher TMAO at three months correlated with an increased risk for new-onset AF at 12 months.

CMD is a poorly understood syndrome that ultimately leads to myocardial ischemia. There are multiple proposed etiologies including oxidative stress leading to a chronic inflammatory response, microthrombi formation, endothelial and vascular smooth muscle dysfunction leading to inappropriate coronary microvascular constriction and/or impaired dilatation [17]. Further, the invasive testing required to definitively diagnose CMD makes it a difficult syndrome to study. TMAO is an emerging biomarker, derived from the enteric system and systemically absorbed, and is thought to play a pro-inflammatory role in the body. Elevated TMAO levels have been associated with an increased risk of coronary artery disease including STEMI, ischemic stroke, and peripheral artery disease [18,19,20,21]. Our study represents the first study to show that higher TMAO levels correlate with the presence of CMD. One prior study examined patients with inflammatory bowel disease and found that gut TMAO concentrations negatively correlated with flow-mediated vasodilatation and coronary flow velocity reserve [22]. However, these are non-invasive parameters which, while suggestive of CMD, are not diagnostic for CMD. The exact mechanism by which TMAO contributes to CMD is still unclear, but it has been proposed that TMAO may disrupt normal endothelial function within the microvasculature, impairing the homeostatic regulation of blood flow [22].

We found that the presence of CMD was also associated with higher BNP and lower creatinine clearance. Chronic kidney disease is associated with microvascular dysfunction in multiple organ systems, including the eye and the brain [23]. The Women’s Ischemia Syndrome Evaluation (WISE) study enrolled women undergoing angiography for suspected ischemia [24]. They found that renal function correlated with CFR even after adjusting for age, diabetes, hypertension, heart rate, hyperlipidemia, BMI, hormone replacement therapy, and the severity of obstructive CAD [25]. This was particularly true in older women, suggesting that chronic kidney disease may contribute to microvascular dysfunction in the myocardium, in addition to other organ systems.

A lower LVEF and higher LAVI correlated with an increased risk of new-onset AF, which is consistent with prior studies [4]. We found that a higher IMR was an independent predictor of new-onset AF, suggesting that CMD contributes to adverse cardiac electrical remodeling, which is consistent with recent literature. Corban et al. performed a long-term study on patients with chest pain and nonobstructive coronary artery disease [26]. They assessed for coronary endothelial dysfunction and found that over a 10.5-year follow-up period, the patients with endothelial dysfunction had a higher risk for new-onset AF (OR 3.87; 95% CI, 1.27–47.0), despite no differences in baseline LVEF or LAVI. Notably, endothelial dysfunction was associated with a higher risk for AF than LAVI. Another study by Ozcan et al. examined patients with heart failure with preserved ejection fraction and found that the presence of CMD was highly correlated with AF (OR 4.38, *p* = 0.02) [27].

Similarly, research has suggested that patients with STEMI are more likely to have CMD and new-onset AF than those without STEMI, although the exact reasons for this are not yet fully understood [28]. CMD is associated with chronic inflammation, oxidative stress and ischemia, which can damage the myocardium and lead to arrhythmias due to adverse cardiac electric remodeling [29,30].

CMD has been linked to heart failure with preserved ejection fraction in recent years [31,32,33]. However, we found that patients with CMD had a reduction in LVEF at 12 months, while patients without CMD had a small increase in LVEF over the same time period. While heart failure with reduced ejection fraction due to ischemic cardiomyopathy is traditionally thought to occur from hypokinetic and/or akinetic myocardium due to scar formation, a recent study by Rahman et al. shows that patients with CMD also have inducible myocardial ischemia on perfusion imaging during exercise [34]. Together, this suggests that acute myocardial infarction patients with CMD may see a reduction in LVEF due to chronic, ongoing ischemia in addition to myocardia scar formation. This gradual reduction in LVEF has also been observed in hypertrophic cardiomyopathy patients with CMD [35].

## 5. Limitations

The study’s primary limitation is that it was conducted at a single institution. Additionally, despite the small size of the study’s sample (200 patients), it is still one of the largest studies involving invasive testing of CMD. Further, since coronary physiology measurements were only obtained after the STEMI event, we were unable to disqualify patients with CMD prior to the event. Furthermore, we were unable to determine if our findings also apply to single-vessel diseased patients because the study recruited only those with a coronary multivessel disease with the intent of obtaining the coronary physiology measurements during the staged PCI. The LVEF was used as a metric for cardiac structural remodeling in this study, but other echocardiographic parameters, such as LV strain and diastolic parameters, were not accounted for. Further, we were unable to control for the ischemia time or the myocardial volume at risk which likely differ across patients and would influence the post-PCI LVEF.

## 6. Conclusions

Patients with STEMI have a relatively high prevalence of CMD when assessed three months after their event. They also have a higher TMAO levels, an emerging biomarker that may play an important role in microvascular disease. Patients with CMD exhibited an increased incidence of new-onset AF at 12 months compared to patients without CMD. Further, the LVEF was lower at 12 months in patients with CMD compared to their post-PCI LVEF, suggesting that the chronic ischemia and adverse cardiac remodeling propagate systolic dysfunction in this population. While this is one of the largest prospective cohorts examining CMD to date, further research is needed to better understand how CMD impacts myocardial recovery in acute myocardial infarction patients and which therapies can ameliorate it deleterious effects.

## Figures and Tables

**Table 1:** Characteristics of ST-elevation myocardial infarction patients classified by coronary microvascular dysfunction.

Characteristic	Overall (n = 200)	No CMD (n = 154)	CMD (n = 46)	*p*-Value
Sex (female)	81 (40.5%)	50 (32.5%)	31 (67.4%)	<0.001
Age (years)	65.5 [58, 76]	67 [58, 76]	63 [57, 76]	0.558
Body mass index (kg/m^2^)	27.53 [24.56, 30.82]	27.64 [24.67, 31.21]	26.75 [24.44, 29.41]	0.319
Body surface area (m^2^)	1.93 [1.81, 2.10]	1.94 [1.81, 2.12]	1.89 [1.83, 2.05]	0.471
Arterial hypertension	118 (59.0%)	93 (60.4%)	25 (54.3%)	0.465
History of coronary artery disease	55 (27.5%)	41 (26.6%)	14 (30.4%)	0.611
History of PCI	24 (12.0%)	17 (11.0%)	7 (15.2%)	0.444
History of stroke	25 (12.5%)	19 (12.3%)	6 (13.0%)	0.899
History of diabetes mellitus	49 (24.5%)	28 (18.2%)	21 (45.7%)	<0.001
History of dyslipidemia	114 (57.0%)	88 (57.1%)	26 (56.5%)	0.940
Smoker (former/current)	108 (54.0%)	78 (50.6%)	30 (65.2%)	0.082
History of alcohol abuse	19 (9.5%)	15 (9.7%)	4 (8.7%)	0.832
Baseline CHADS2-VASc score	3 [2, 4]	3 [2, 4]	4 [3, 5]	0.038
KILLIP class				0.944
I	59 (29.5%)	46 (29.9%)	13 (28.3%)	
II	106 (53.0%)	80 (51.9%)	26 (56.5%)	
III	26 (13.0%)	21 (13.6%)	5 (10.9%)	
IV	9 (4.5%)	7 (4.5%)	2 (4.3%)	

CMD = Coronary microvascular dysfunction; PCI = Percutaneous Coronary Intervention.

**Table 2:** Laboratory tests of patients with ST-elevation myocardial infarction during admission and follow-up, categorized by CMD.

Laboratory Test	Overall (n = 200)	No CMD (n = 154)	CMD (n = 46)	*p*-Value
On admission				
Hemoglobin (g/L)	135.50 [119, 147]	135 [119, 146]	141.5 [115, 148]	0.983
Red cell distribution width (%)	13.70 [13.10, 14.40]	13.70 [13.1, 14.4]	13.65 [13.20, 14.50]	0.962
White Blood Cell Count (10^9^/L)	9.83 [8.09, 12.10]	9.76 [8.07, 11.98]	10.42 [8.16, 12.99]	0.372
Neutrophil (10^9^/L)	7.09 [5.29, 9.0]	7.09 [5.20, 8.85]	6.96 [5.60, 9.61]	0.533
Lymphocyte (10^9^/L)	1.85 [1.28, 2.61]	1.85 [1.31, 2.62]	1.83 [1.27, 2.59]	0.825
Neutrophil-Lymphocyte Ratio	3.81 [2.34, 5.74]	3.80 [2.35, 5.77]	3.87 [2.33, 5.49]	0.938
Platelets (×10^9^/L)	240 [203, 272.50]	239.5 [203, 265]	241.5 [200, 283]	0.628
Total cholesterol (mmol/L)	4.67 [3.77, 5.84]	4.71 [3.84, 5.90]	4.53 [3.54, 5.60]	0.449
Low-density lipoprotein (mmol/L)	3.02 [2.35, 4.13]	3.04 [2.37, 4.15]	2.98 [2.28, 4.09]	0.780
High-density lipoprotein (mmol/L)	1.11 [0.92, 1.36]	1.13 [0.95, 1.36]	1.09 [0.88, 1.32]	0.390
Triglycerides (mmol/L)	1.17 [0.82, 1.68]	1.18 [0.82, 1.69]	1.14 [0.88, 1.57]	0.671
Creatinine Clearance (mL/min)	39.5 [34.8, 47.5]	40.9 [36.10, 48.60]	36.6 [32.80, 42.50]	0.001
Basal Troponin I (µg/L)	2.21 [0.81, 3.88]	2.16 [0.77, 3.58]	2.23 [0.89, 4.77]	0.179
Peak Troponin I (µg/L)	45.0 [27.0, 65.0]	42.0 [27.00, 62.00]	50.0 [28.00, 81.00]	0.140
High-sensitivity C-reactive protein (mg/L)	3.66 [1.85, 10.52]	4.77 [2.36, 11.89]	2.23 [1.36, 5.82]	0.005
During follow-up				
Hemoglobin (g/L)	142 [134, 153]	142 [133, 152]	141 [136, 153]	0.477
Red cell distribution width (%)	13.50 [12.90, 14.50]	13.50 [12.80, 14.60]	13.4 [13.0, 14.20]	0.760
White Blood Cell Count (10^9^/L)	9.30 [7.40, 11.99]	9.50 [7.42, 11.81]	9.15 [7.16, 13.32]	0.941
Platelets (×10^9^/L)	209 [166.5, 249.0]	209 [168, 249]	208.5 [165, 257]	0.785
Total cholesterol (mmol/L)	3.25 [2.87, 4.17]	3.19 [2.84, 3.95]	3.86 [2.97, 4.91]	0.005
Low-density lipoprotein (mmol/L)	2.12 [1.51, 2.63]	2.12 [1.47, 2.45]	2.15 [1.81, 3.40]	0.014
High-density lipoprotein (mmol/L)	1.25 [1.12, 1.48]	1.25 [1.12, 1.52]	1.30 [1.12, 1.42]	0.658
Triglycerides (mmol/L)	1.11 [0.74, 1.47]	1.11 [0.74, 1.49]	1.06 [0.78, 1.25]	0.675
High-sensitivity C-reactive protein (mg/L)	4.21 [2.29, 6.30]	4.02 [1.98, 6.14]	4.67 [3.27, 6.87]	0.021
Erythrocyte sedimentation rate (mm/h)	13.0 [9.0, 17.0]	12.50 [9, 16]	15.50 [10, 22]	0.040
B-type natriuretic peptide (ng/L)	37.0 [27.0, 66.75]	37.0 [27.0, 51.0]	73.0 [29.0, 104.0]	<0.001
Trimethylamine N-oxide (µM)	2.96 [1.82, 4.56]	2.41 [1.34, 3.54]	5.28 [3.97, 7.45]	<0.001

CMD = Coronary microvascular dysfunction.

**Table 3:** Angiographic, echocardiographic, and physiologic parameters of ST-elevation myocardial infarction patients during admission and follow-up, characterized by coronary microvascular dysfunction.

Parameters	Overall (n = 200)	No CMD (n = 154)	CMD (n = 46)	*p*-Value
Angiographic.				
Pain-to-door time (minutes)	314 [107.75, 597.0]	314 [104, 503]	373 [141, 767]	0.051
Door-to-balloon (minutes)	40 [30, 51.75]	40 [29.0, 51.0]	40.5 [33.0, 55.0]	0.323
Pre-PCI TIMI flow				0.258
0	124 (62.0%)	90 (58.4%)	34 (73.9%)	
1	8 (4.0%)	6 (3.9%)	2 (4.30%)	
2	40 (20.0%)	34 (22.1%)	6 (13.0%)	
3	28 (14.0%)	24 (15.6%)	4 (8.7%)	
Post-PCI TIMI flow				0.259
0	2 (1.0%)	1 (0.6%)	1 (2.2%)	
1	1 (0.5%)	0 (0.0%)	1 (2.2%)	
2	20 (10.0%)	15 (9.7%)	5 (10.9%)	
3	177 (88.5%)	138 (89.6%)	39 (84.8%)	
Culprit Vessel				0.068
Left anterior descending artery	111 (55.5%)	86 (55.8%)	25 (54.3%)	
Circumflex artery	49 (24.5%)	42 (27.3%)	7 (15.2%)	
Right coronary artery	40 (20.0%)	26 (16.9%)	14 (30.4%)	
Number of diseased vessels				0.913
2-Vessel Disease	116 (58.0%)	89 (57.8%)	27 (58.7%)	
3-Vessel Disease	84 (42.0%)	65 (42.2%)	19 (41.3%)	
Echocardiographic				
Post-PCI left atrial diameter (mm)	36.0 [32.0, 38.75]	35.0 [32.0, 38.0]	37.0 [34.0, 40.0]	0.183
Post-PCI left atrial Volume (mL)	50.0 [45.25, 62.75]	50.0 [46.0, 57.0]	53.0 [44.0, 75.0]	0.209
Post-PCI left atrial volume index (mL/m^2^)	26.33 [22.62, 31.86]	26.24 [22.60, 31.25]	26.58 [23.02, 39.54]	0.267
Post-PCI left ventricular ejection fraction (%)	40.0 [36.0, 45.0]	40.0 [35.0, 45.0]	45.0 [40.0, 50.0]	0.019
12-month left ventricular ejection fraction (%)	45.0 [40.0, 50.0]	50.0 [40.0, 55.0]	40.0 [32.0, 40.50]	<0.001
Coronary physiology at 3-month follow-up				
Coronary flow reserve	2.68 [2.19, 2.90]	2.84 [2.64, 2.98]	1.85 [1.32, 2.17]	<0.001
Fractional flow reserve	0.92 [0.87, 0.97]	0.92 [0.87, 0.97]	0.90 [0.85, 0.94]	0.116
Index of microvascular resistance	20 [14.25, 28.25]	18.0 [13.0, 21.0]	44.0 [38.0, 51.0]	<0.001

CMD = Coronary microvascular dysfunction; PCI = Percutaneous Coronary Intervention.

**Table 4:** Clinical and Echocardiographic outcomes by coronary microvascular dysfunction.

Outcome	Overall (n = 200)	No CMD (n = 154)	CMD (n = 46)	*p*-Value
New-onset of atrial fibrillation	22 (11.0%)	7 (4.5%)	15 (32.6%)	<0.001
LVEF improvement	154 (78.6%)	141 (92.8%)	13 (29.5%)	<0.001

LVEF = Left ventricular ejection fraction.

**Table 5:** Adjusted and unadjusted univariate binary logistic regression model for prediction of atrial fibrillation event.

	Unadjusted Univariable Analysis				Adjusted Univariable Analysis	
Variable	Odds Ratio (95% CI)	AUC	AIC	*p*-Value	Odds Ratio (95% CI)	*p*-Value
Sex (female)	2.899 (1.155–7.276)	0.630	137.223	0.023		
Age	1.003 (0.967–1.041)	0.503	142.358	0.867		
Body mass index	0.890 (0.798–0.993)	0.353	137.470	0.038		
Inferior STEMI	0.654 (0.261–1.637)	0.449	141.761	0.364	0.627 (0.242–1.624)	0.336
Arterial hypertension	1.004 (0.408–2.472)	0.501	142.606	0.993	0.979 (0.385–2.491)	0.965
History of CAD	0.753 (0.264–2.150)	0.473	142.314	0.596	0.768 (0.259–2.279)	0.635
History of stroke	1.120 (0.306–4.095)	0.506	142.578	0.864	0.910 (0.240–3.450)	0.889
History of diabetes mellitus	2.388 (0.952–5.993)	0.592	139.346	0.064	1.897 (0.713–5.045)	0.200
History of dyslipidemia	2.177 (0.814–5.821)	0.588	139.999	0.121	2.094 (0.758–5.789)	0.154
Smoker (former/current)	1.959 (0.762–5.035)	0.580	140.551	0.163	1.767 (0.665–4.699)	0.254
Baseline CHADS2-VASc score	1.112 (0.825–1.501)	0.549	142.123	0.486	0.853 (0.546–1.332)	0.485
Pain-to-door time	1.001 (1.000–1.001)	0.587	137.321	0.015	1.001 (1.000–1.001)	0.041
Door-to-balloon	0.992 (0.966–1.019)	0.472	142.243	0.554	0.992 (0.964–1.020)	0.558
Basal Troponin I	1.071 (1.008–1.139)	0.576	138.013	0.026	1.102 (1.029–1.180)	0.005
Creatinine Clearance	0.965 (0.919–1.013)	0.380	140.370	0.154	0.966 (0.923–1.010)	0.130
Neutrophil-Lymphocyte Ratio	0.944 (0.802–1.111)	0.493	142.002	0.486	0.970 (0.818–1.152)	0.731
3-month Total cholesterol	1.443 (1.034–2.013)	0.678	138.283	0.031	1.383 (0.948–2.017)	0.092
3-month LDL	1.657 (1.075–2.556)	0.677	137.713	0.022	1.521 (0.938–2.466)	0.089
3-month HDL	0.543 (0.121–2.432)	0.448	141.937	0.425	0.439 (0.087–2.208)	0.318
3-month Triglycerides	1.072 (0.589–1.953)	0.565	142.555	0.820	1.114 (0.597–2.079)	0.734
3-month High-sensitivity CRP	0.995 (0.974–1.016)	0.470	142.306	0.623	1.011 (0.857–1.192)	0.897
3-month ESR	1.085 (1.014–1.161)	0.622	137.101	0.019	1.076 (1.005–1.152)	0.036
3-month BNP	1.007 (1.001–1.012)	0.607	137.150	0.014	1.005 (0.999–1.011)	0.085
3-month TMAO	1.216 (1.055–1.401)	0.669	135.771	0.007	1.200 (1.031–1.397)	0.018
3-Vessel Disease	0.767 (0.306–1.921)	0.468	142.280	0.571	0.800 (0.310–2.065)	0.645
Post-PCI left atrial diameter	1.159 (1.082–1.242)	0.823	122.310	<0.001	1.206 (1.107–1.313)	<0.001
Post-PCI LAVI	1.211 (1.128–1.299)	0.914	91.959	<0.001	1.225 (1.136–1.322)	<0.001
Post-PCI LVEF (%)	0.981 (0.922–1.045)	0.472	142.261	0.554	0.984 (0.925–1.046)	0.599
Coronary flow reserve	0.385 (0.199–0.746)	0.265	134.287	0.005	0.475 (0.234–0.963)	0.039
Fractional flow reserve	0.004 (0.001–9.907)	0.433	140.702	0.167	0.001 (0.000–4.362)	0.110
IMR	1.057 (1.029–1.085)	0.763	125.668	<0.001	1.050 (1.021–1.081)	0.001

AUC = area under the receiver operating characteristic curve; CI = confidence interval; AIC = Akaike information criterion; STEMI = ST elevation myocardial infarction; CAD = coronary artery disease, LDL = Low-density lipoprotein, HDL = High-density lipoprotein, CRP = High-sensitivity C-reactive protein, ESR = Erythrocyte sedimentation rate, BNP = B-type natriuretic peptide, TMAO = Trimethylamine N-oxide LAVI = left atrial volume index, LVEF = left ventricular ejection fraction, IMR = Index of microvascular resistance.

**Table 6:** Adjusted multivariate binary logistic analysis for prediction of atrial fibrillation event.

Effect	Odds Ratio	95% Confidence Limits		*p*-Value
Post-PCI left ventricular ejection fraction (%)	0.897	0.813	0.989	0.029
Post-PCI left atrial volume index (mL/m^2^)	1.261	1.146	1.387	<0.001
Trimethylamine N-oxide (µM) Index of microvascular resistance	1.290 1.066	1.002 1.018	1.660 1.117	0.048 0.007

PCI = Percutaneous Coronary Intervention.

**Table 7:** Multivariable model for prediction of left ventricular ejection fraction improvement.

Effect	Odds Ratio	95% Confidence Limits		*p*-Value
Coronary flow reserve	2.438	1.064	5.585	0.035
Index of microvascular resistance	0.941	0.910	0.973	<0.001
Trimethylamine N-oxide (µM)	0.777	0.665	0.909	0.002

## Data Availability

The datasets used in this study are available from the corresponding author upon reasonable request.
